# Performance of Carbon Nanotube/Polysulfone (CNT/Psf) Composite Membranes during Oil–Water Mixture Separation: Effect of CNT Dispersion Method

**DOI:** 10.3390/membranes7010014

**Published:** 2017-03-06

**Authors:** Michael Olawale Daramola, Palesa Hlanyane, Oluwafolakemi O. Sadare, Olugbenga O. Oluwasina, Sunny E. Iyuke

**Affiliations:** 1 School of Chemical and Metallurgical Engineering, Faculty of Engineering and the Built Environment, University of the Witwatersrand, Johannesburg, Private Bag 3, Wits 2050, South Africa; palesa.hlanyane@sasol.com (P.H.); wumioladejo@yahoo.com (O.O.S.); Sunny.Iyuke@wits.ac.za (S.E.I.); 2 Department of Chemistry, School of Sciences, Federal University of Technology, Akure 220001, Nigeria; oooluwasina@futa.edu.ng

**Keywords:** carbon nanotubes, composite membranes, mixed matrix membrane, separation, oily wastewater

## Abstract

Effect of the dispersion method employed during the synthesis of carbon nanotube (CNT)/polysulfone-infused composite membranes on the quality and separation performance of the membranes during oil–water mixture separation is demonstrated. Carbon nanotube/polysulfone composite membranes containing 5% CNT and pure polysulfone membrane (with 0% CNT) were synthesized using phase inversion. Three CNT dispersion methods referred to as Method 1 (M1), Method 2 (M2), and Method 3 (M3) were used to disperse the CNTs. Morphology and surface property of the synthesized membranes were checked with scanning electron microscopy (SEM) and Fourier-transform infrared (FTIR) spectroscopy, respectively. Separation performance of the membranes was evaluated by applying the membrane to the separation of oil–water emulsion using a cross-flow filtration setup. The functional groups obtained from the FTIR spectra for the membranes and the CNTs included carboxylic acid groups (O–H) and carbonyl group (C=O) which are responsible for the hydrophilic properties of the membranes. The contact angles for the membranes obtained from Method 1, Method 2, and Method 3 were 76.6° ± 5.0°, 77.9° ± 1.3°, and 77.3° ± 4.5°, respectively, and 88.1° ± 2.1° was obtained for the pure polysulfone membrane. The oil rejection (OR) for the synthesized composite membranes from Method 1, Method 2, and Method 3 were 48.71%, 65.86%, and 99.88%, respectively, indicating that Method 3 resulted in membrane of the best quality and separation performance.

## 1. Introduction

Application of mixed matrix membranes (MMMs) in wastewater treatment is gaining interest amongst researchers due to the enhanced mechanical strength, permeability, and selectivity of these membranes when compared to those of equivalent pure polymeric membranes [1]. MMMs are prepared by dispersing porous particles, such as zeolites [2], carbon molecular sieves [3], silica [4], and carbon nanotubes (CNTs) [5,6] within the polymer matrix of polymers such as polysulfone, polyether sulfone, and polyamide [1].

Kumar et al. [7] studied the performance of polysulfone (Psf)–bentonite ultrafiltration membrane and polysulfone–silica membrane for the purification of oil-containing produced water. The membranes were prepared via phase inversion technique by blending silica and bentonite nanoparticles separately with polysulfone and *N*-methyl-2-pyrrolidone solution. The result of effects of silica- and bentonite-loading on pure water flux at different transmembrane pressure (TMP) (100–300 kPa) at an increment of 50 kPa for pure water flux for membrane Psf (pure without nanoparticles), Psf/silica-3 (containing 3 wt % silica), Psf/silica-8 (containing 8 wt % silica), Psf/bentonite-3 (containing 3 wt % bentonite), and Psf/bentonite-8 (containing 8 wt % bentonite) were 60, 100, 140, 122, and 309 L·m^−2^·h^−1^ at 1 bar, respectively. The permeability recorded for these membranes were 0.97 × 10^10^ m·Pa^−1^·s^−1^ (for Psf), 1.53 × 10^10^ m·Pa^−1^·s^−1^ (for Psf-silica-3), 2.42 × 10^10^ m·Pa^−1^·s^−1^ (Psf-silica-8), 2.11 × 10^10^ m·Pa^−1^·s^−1^ (Psf/bentonite-3), and 5.25 × 10^10^ m·Pa^−1^·s^−1^ (Psf/bentonite-8). The authors concluded that addition of bentonite nanoparticles to Psf is a very effective strategy to increase the water flux of the membranes when compared to that of the Psf–silica membranes; the increase in pure water flux in Psf/bentonite membranes was attributed to the enhancement of the hydrophilicity of the membranes. For the separation of oil from synthetic oil/water mixture (oil concentration 200 mg·L^−1^), results of oil rejection ratio (OR) showed that the OR trend decreased as nanoparticle loading increased up to 8 wt %. However, for almost all the membranes, the OR was >90%.

In the same vein, Shu et al. [8] developed composite membranes with an asymmetric three-layer structure (i.e., a porous ceramic membrane substrate), a polyvinylidene fluoride (PVDF) ultrafiltration sublayer, and a polyamide/polyvinyl alcohol (PVA) composite thin top-layer, for the purification of oil-containing water microemulsions. The results revealed that at the operating pressure of 0.4 MPa, the hydraulic permeability was about 190 L/m^2^h with OR > 98.5%.

In a recent study reported by Maphutha et al. [9], synthesis of MMM containing polysulfone as the substrate and CNTs as the fillers for the treatment of oil-containing water was studied. To further enhance the fouling resistance, the selectivity, and the mechanical strength of the membranes, the top layer of the MMM was covered with a thin layer of polyvinyl alcohol (PVA). The membrane was applied to the separation of oil–water mixture and the authors reported oil rejection >90% [9]. According to the authors, the presence of the PVA layer enhanced the membrane selectivity, but the membrane with the PVA layer displayed a dramatic decrease in water flux due to an increase in the wall thickness of the membrane. In addition, the use of the PVA constitutes an additional operating cost to the membrane fabrication.

Furthermore, weak interaction of the dispersed fillers within the polymer matrix in MMMs has been identified as one of the shortcomings of these types of membranes [2]. The weak interaction results in the creation of large defects (i.e., large free fraction volume (FFV) between the fillers and the polymer matrix) that could promote loss in membrane selectivity. The weak interaction of the fillers within the polymer could be attributed to the poor dispersion of the filler in the polymer matrix phase during the synthesis and the size of the fillers [2]. The aforementioned problems could have detrimental effects on the quality of MMMs [9], resulting in poor separation performance. Other challenges encountered with the application of MMMs in wastewater treatment are fouling and concentration polarization [9], but surface modification by functionalizing the fillers and surface of the MMMs have been proposed as possible measures to overcome the challenges [5,6,7,8,9]. However, in the case of poor dispersion of the fillers within the polymer materials, especially for carbon nanotube/polysulfone (CNT/Psf) composite membranes, limited reports have been documented in open literature. In a recent study by Maphutha and his coworkers, in which the synthesis of carbon nanotube/polysulfone/polyvinyl alcohol (CNT/Psf/PVA) composite membranes has been reported for the separation of oil-containing wastewater, the CNTs were dispersed within the polysulfone using mechanical stirring. During the preparation, Psf was first dissolved in the solvent, followed by the addition of CNTs. The mixture was stirred for 24 h before hand-casting. The performance evaluation of the membranes during oil-containing wastewater treatment resulted in oil rejection of 96% [9]. Producing highly selective MMMs depends on the degree of the dispersion of the fillers within the matrix of the support polymer. Different dispersion methods will result in MMMs of different degrees of quality displaying different performances during the separation of oil–water emulsion [1]. Against this background and as a follow-up to the work of Maphutha and his coworkers, this study investigated the effect of dispersion of CNTs on the quality and performance of CNTs/polysulfone composite membranes during the separation of oil–water emulsion. In addition, the study considered the synthesis of the composite membranes without adding the polyvinyl alcohol (PVA) layer to evaluate its effect on the membrane quality and performance.

## 2. Experimental

### 2.1. Materials

Multiwalled carbon nanotubes (outside diameter (OD) 6–9 nm × L 5 μm and carbon basis greater than 90%), polysulfone (Psf) (in beaded form with a molecular weight of 22,000 g/mol), and *N*,*N*-dimethylformamide (DMF) (solvent with molecular weight of 73.09 g/mol) used in the study were purchased from Sigma Aldrich (Johannesburg South Africa). The materials were used as delivered without any purification, but the CNTs were characterized to confirm the purity.

### 2.2. Membrane Synthesis

**Figure 1:**
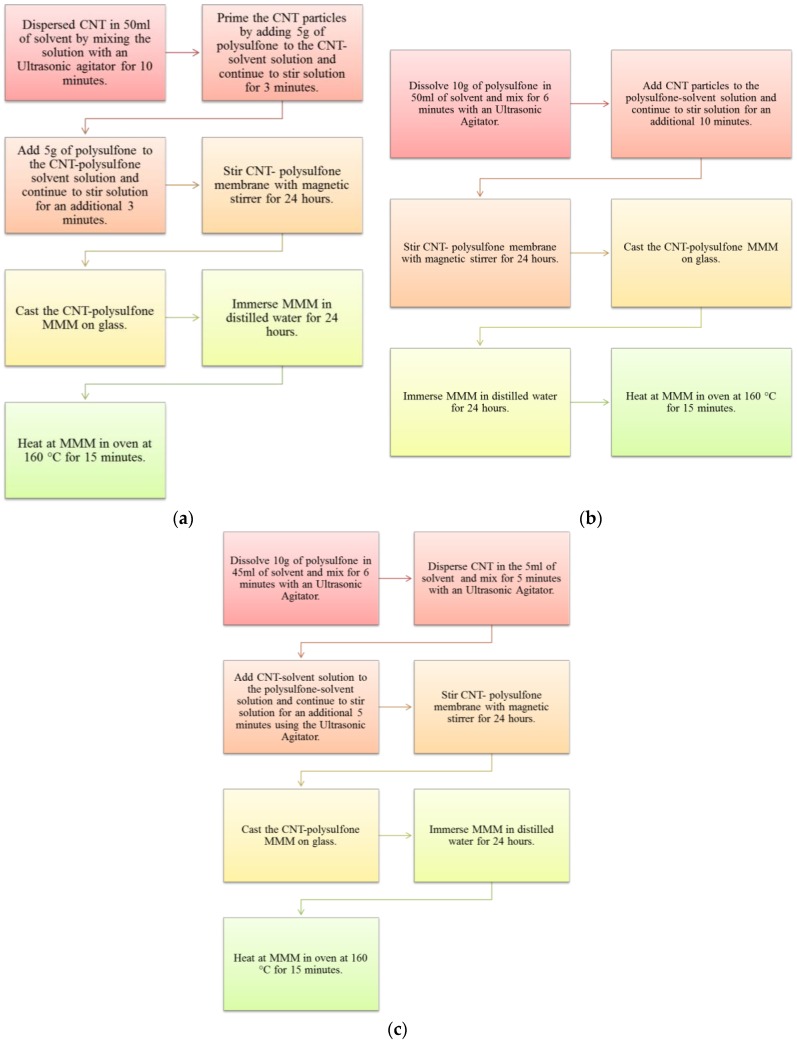
Dispersion methods employed during the synthesis of the carbon nanotube/polysulfone (CNT/Psf) composite membranes. Method 1 (M1) (**a**); Method 2 (M2) (**b**); and Method 3 (M3) (**c**) (Adapted from reference [1]).

The CNT dispersion methods employed in the synthesis of the membranes were adopted from reference [1] and are referred to as Method 1, Method 2, and Method 3 in this article. The membranes obtained from Method 1, Method 2, and Method 3 are also referred to as M1, M2, and M3 in this study. For Method 1, CNT was first dispersed in DMF by stirring for 10 min using an ultrasonicator, followed by the addition of some Psf into the homogeneous suspension, and mixing for 3 min. The mixture was stirred for another 24 h to attain homogeneity before hand-casting on a glass plate using a casting device called “Dr Blade”, followed by phase inversion procedure (see Figure 1a). In Method 2, Psf was first dissolved in the DMF and mixed for a 1 h, then followed by the addition of CNT, and further mixed for another 30 min. Then, the mixture was stirred for another 24 h to attain homogeneity before hand-casting on a glass plate followed phase inversion procedure as in M1 (see Figure 1b). In Method 3, two homogeneous solutions—Solution A and Solution B—were prepared separately and later mixed. Solution A contained dispersed CNT particles in DMF and Solution B contained dissolved Psf in DMF. Solution A and Solution B were then mixed together and stirred for 30 min, and then for another 24 h until homogeneity was attained. Hand-casting and phase inversion procedures in M3 were as explained in M1 and M2 (see Figure 1c). The mass percentage of the CNTs in all the precursor solution was 5 wt %. For comparison, pure Psf membrane (M4, with 0% CNTs) was prepared by dissolving polysulfone in the DMF, stirring for 24 h until homogeneity was attained, and fabricating the membrane as described for M1, M2, and M3. The MMMs obtained after the casting were heated in oven at 160 °C to get rid of the solvent completely. The supplied CNTs and the membranes were characterized using SEM and TEM (for CNTs) for morphology, Fourier-transformation infrared (FTIR) for surface chemistry, and contact angle measurement for hydrophilicity of the membranes. The as-received CNTs from the supplier were characterized to confirm the report included in the supply by the supplier.

### 2.3. Performance Evaluation of the Synthesized Membranes

**Figure 2:**
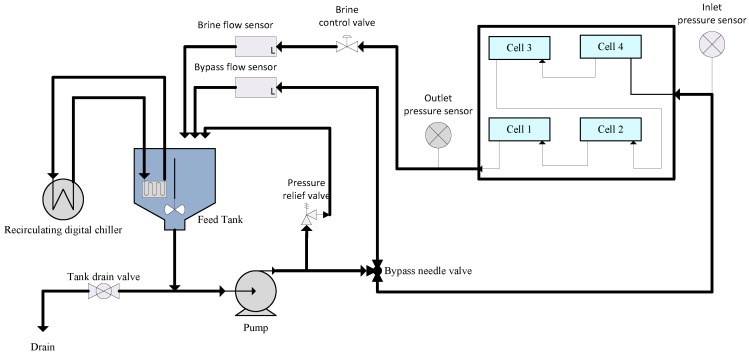
Process flow diagram of the Sterlitech cross-flow filtration module.

The performance of the synthesized membranes during oil-containing wastewater separation was evaluated using a synthetic oil–water mixture (diesel, with components generally from the C_8_ to C_21_ alkane groups in water emulsion) and a Sterlitech cross-flow filtration (see Figure 2 for the process flow diagram of the Sterlitech cross-flow filtration). The Sterlitech cross-flow filtration module consisted of a feed tank, pump, four filtration cells, a chiller unit, and flow and pressure controllers. A high-speed stirrer was used in the feed tank to maintain a homogeneous emulsion of the oil and water mixture. A pump was used to transfer oil–water mixture from the feed tank to the filtration cells, where the membranes were fitted during the separation. The module has four filtration cells that were used during the separation. Permeate from the cell was recirculated back to the tank via flexible hose.

Pure water flux (PWF), the oily water flux (OWF), and the oil rejection ratio (OR) at various pressures were evaluated and used as performance indicators. PWF, OWF, the permeability (oil–water permeability per unit thickness of the membrane (OWP) and pure water permeability per unit thickness of the membrane (PWP)) and OR were determined using Equations (1), (2), and (4), respectively:

(1) $$F=\frac{V}{A}$$

where *F* is the permeate flux (L/m^2^h); *V*, the volumetric flow (L/h); and *A*, the effective membrane area (m^2^).

(2) $$P=\frac{F}{TMP}$$

where *P* is the permeability per unit thickness of the membrane and *TMP* is the transmembrane pressure (bar) obtained from Equation (3):

(3) $$TMP=\frac{P_{1}+P_{2}}{2}-P_{p}$$

where *P*_1_ and *P*_2_ are the upstream (feed) and downstream (retentate) pressure of the membrane in the cross-flow module cell expressed in bar, respectively, and $P_{p}$.

(4) $$OR=\frac{\left(C_{F}-C_{P}\right)}{C_{F}}\times 100\left(\%\right)$$

where *OR* is the membrane oil rejection ratio expressed in percentage; $C_{F}$ is the oil concentration in the feed stream to the cross-flow unit (mg/L); and $C_{P}$ is the oil concentration in the permeate stream from the cross-flow cell (mg/L).

## 3. Results and Discussion

### 3.1. Characterization of the CNTs and the Fabricated Membranes

**Figure 3:**
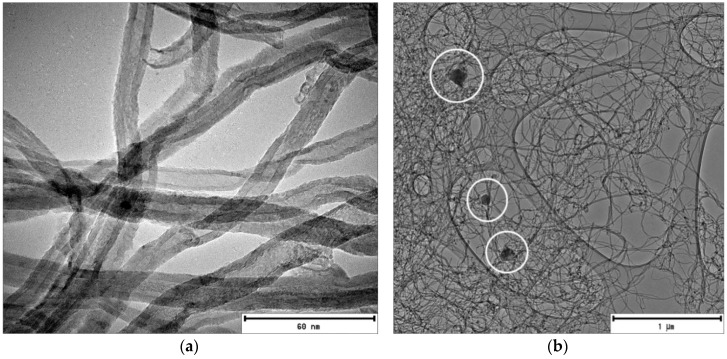
TEM micrograph of CNT supplied by Sigma Aldrich. (**a**) Indicating the morphology of the CNTs at high magnification; (**b**) the white encircled areas indicate lumpy material adhering to the CNTs.

The morphology of the supplied CNT samples was examined using SEM and TEM using the FEI Technai TEM operated at a voltage of 120 kV as used in the previous studies [10,11,12] to confirm the specification report that accompanied the supply. The internal diameter of the CNTs was <10 nm and the length >4 μm (see Figure 3a,b). TEM micrographs indicate the presence of dark spots and lumpy material adhering on the surface of the CNTs encircled in Figure 3b. Similar morphology has been reported, and the lumpy material was identified as particle impurities, usually in the form of graphitic, metal catalytic and amorphous carbon, attached on the carbon fiber surface of CNTs during the synthesis process [13]. In addition, the TEM micrographs confirm that the CNT are multiwalled, as specified by the suppliers.

**Figure 4:**
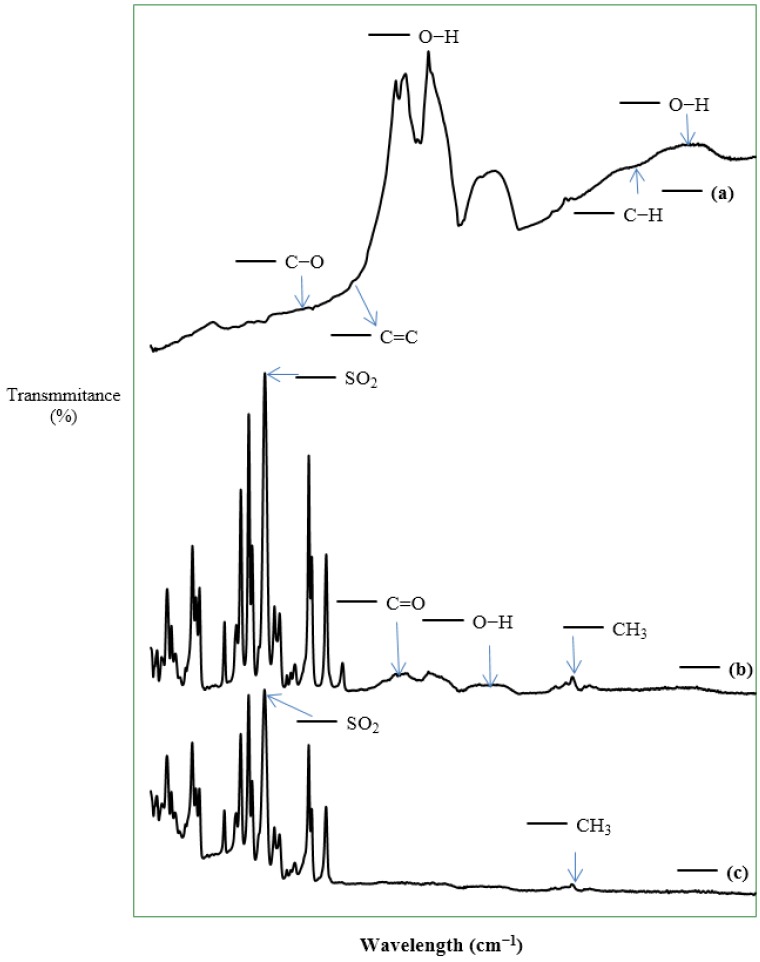
Fourier-transformation infrared (FTIR) spectra showing functional groups. (**a**) CNTs; (**b**) M1; and (**c**) M4.

The FTIR spectra depicted in Figure 4 show the surface functional groups of the supplied CNTs and the fabricated membranes. A peak at 1157 cm^−1^ was observed for the CNTs as shown in Figure 2a, attributable to esterified alcohol (C–O) groups, and a peak of low intensity was observed at 1558 cm^−1^, attributable to C=C double bonding as reported by Pourfayaz et al. [14], which confirms the integrity of the hexagonal structure of multiwalled CNTs [15]. According to Pourfayaz et al. [14], the C=C double bond observed indicates surface defects of CNTs. Peaks, in the range above 3500–3650 cm^−1^, were observed for the CNTs, which have been attributed to stretching vibrations of carboxylic groups (O–H) by McCurry [16]. The observed functional groups, including carboxylic acid (O–H), C–H, alkane groups (C–H), carbonyl compound groups (C=O), and carbon double bonds (C=C) were observed in pristine and functionalized carbon nanotubes by Yudianti et al. [15], confirming that our FTIR results are consistent with literature.

For the membranes, M1 and M4 were examined because M1 is the representative of the synthesized CNT/Psf composite membranes and M4 is the pure polymer membrane. The most important functional groups detected in M4 were sulfur dioxide (SO_2_) and the methyl (CH_3_) group at wavelengths of 1150 cm^−1^ and 2866 cm^−1^, respectively. This observation is consistent with the report of Voicu et al. [17] for polysulfone polymer. For M1, the peak at 1500 cm^−1^ could be attributed to single-bond functional groups. Similar to M4, M1 showed peaks at 1150 cm^−1^ and 1587 cm^−1^, which could be attributed to the stretching vibrations of SO_2_ and aromatic rings, respectively, which form part of the main structural components of the polysulfone polymer. Peaks ranging from 1670 to 1780 cm^−1^ could be attributed to the stretching vibration of carbonyl (C=O) group of the CNTs [15]. In addition, the peaks at 1971 cm^−1^, 2177 cm^−1^, and 2941 cm^−1^ are similar to the ones observed for the CNTs, indicating the presence of CNTs in the fabricated membranes (see Figure 4c). These peaks could be attributed to aromatic rings, carboxylic acid groups (O–H), and alkane groups (C–H) in the membranes [16]. For the M4 membranes, no peaks were at 1971 cm^−1^, 2177 cm^−1^, or 2941 cm^−1^ (see Figure 4b), confirming further the presence of CNTs in the fabricated membranes.

**Figure 5:**
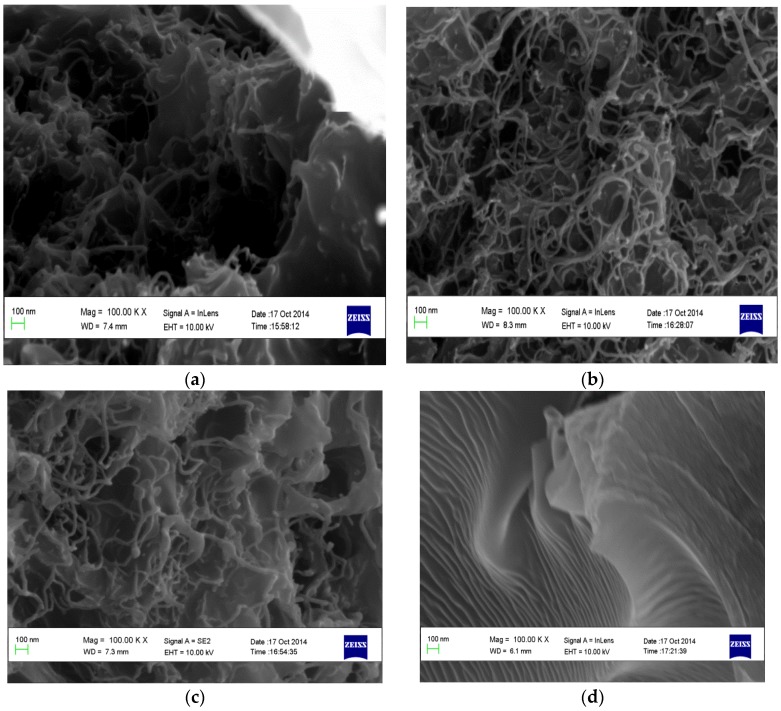
SEM micrographs of the cross-sectional view of the membranes showing their morphologies (**a**) M1; (**b**) M2; (**c**) M3; and (**d**) M4.

**Figure 6:**
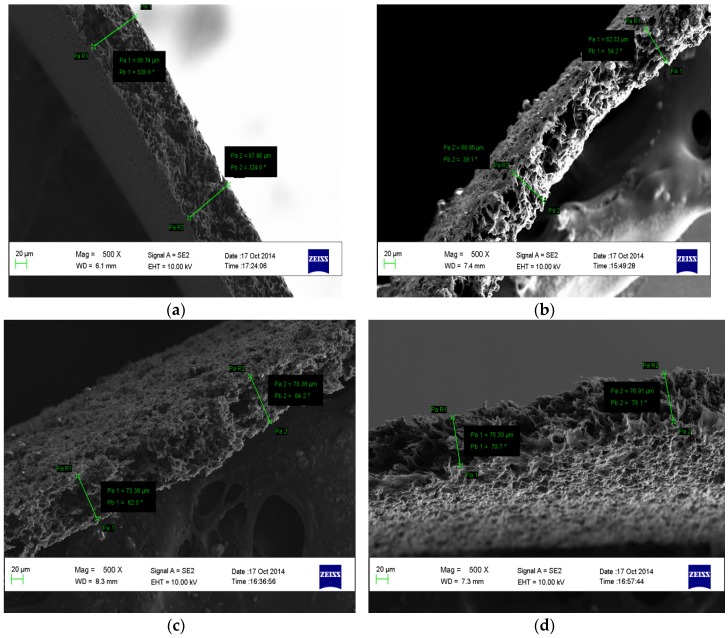
SEM images of the cross-sectional view of the membranes showing their porous structure (**a**) M1; (**b**) M2; (**c**) M3; and (**d**) M4.

The SEM images of the cross-sections of the membranes (M1, M2, M3, and M4) are depicted in Figure 5. The presence of CNTs in M1, M2, and M3 is clearly shown in the images, and the SEM image for the M4 membrane shows a smooth, clear surface (see Figure 5d). In the M1, M2, and M3 membranes, the CNTs appear to be entangled in the membranes in a similar way as indicated by the TEM micrographs of the CNTs. Figure 6 shows the porous structure of the fabricated membranes. This is consistent with literature [7,8,9,18] and the formation of the porous structures could be attributed to rapid mass transfer between water and the solvent in a nonsolvent bath during the synthesis.

### 3.2. Hydrophilicity of the Fabricated Membranes

The contact angles of the membranes were determined using the sessile drop method. The results obtained show that the addition of CNT in the polysulfone matrix results in a decrease of the contact angle, enhancing thereby the hydrophilicity of the membranes (see Table 1). A decrease in contact angle between surface of a material and water is always used as an indication of improvement in the hydrophilic property of the material [19].

Jeong et al. [20] have also noted a decrease in the contact angle of polymeric membranes after the addition of fillers as an indication of an increase in the hydrophilic property of the membranes. Comparison between the synthesized membranes show that M1 is most hydrophilic of the membranes and M4 is the least hydrophilic. In addition, improvement of hydrophilicity of a membrane is one of the ways to mitigate fouling [21]. The results obtained in this study indicate that the CNT/Psf membranes will be less prone to fouling when compared to the pure Psf membranes.

### 3.3. Performance Evaluation of the Membrane

#### 3.3.1. Pure Water Permeation

**Figure 7:**
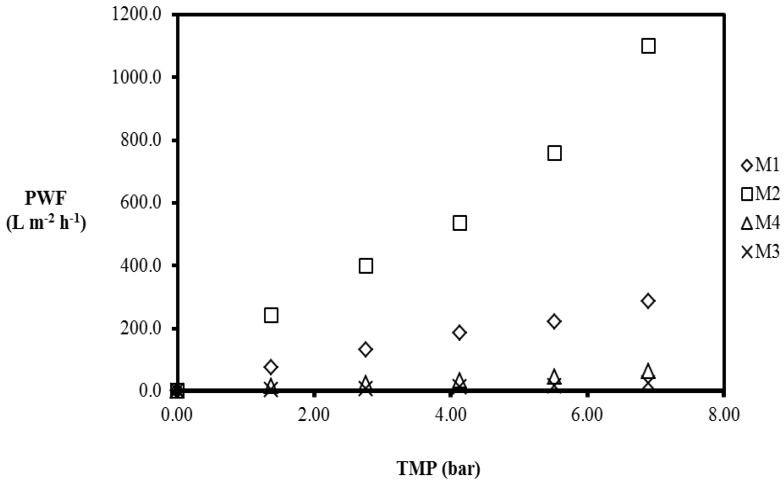
Pure water flux (PWF) of M1, M2, M3 and M4. Experimental conditions: transmembrane pressure (TMP): 1.38–6.9 bar, temperature: room temperature.

During water purification using membranes, pure water flux (PWF) of the membrane is determined to indicate the initial flux of the membrane for fouling monitoring [22]. In this study, PWF of the prepared membranes was evaluated using the Sterlitech cross-flow filtration module at various pressure and the PWF calculated using Equation (1). The effective membrane area used in the evaluation was 42 cm^2^, and the measurement was at room temperature after membrane compaction for 4 h at 8 bar. Deionized water was used for the pure water flux measurements at various transmembrane pressures. Figure 7 depicts the PWF of the membranes.

As can be seen in Figure 7, M4 has the lowest PWF when compared to those of the CNT/Psf membranes. Comparing the CNT/Psf membranes, the PWF of the membranes are in the order: M2 > M1 > M3. In addition, the PWF increased with increasing TMP (see Figure 7). The results obtained in this study are consistent with literature [23]. Furthermore, the pure water permeability (PWP) of the membranes were obtained from Equation (3) [24].

The PWF at 6.9 bar was measured to be 287.7 L·m^−2^·h^−1^, 1098.9 L·m^−2^·h^−1^, 63.3 L·m^−2^·h^−1^, and 25.2 L·m^−2^·h^−1^ for M1, M2, M3, and M4, respectively. M2 displayed the highest PWF for the given TMP range, followed by M1, and M3 displayed the lowest PWF for the MMMs. Increasing the applied pressure enhanced the driving force for permeation, resulting in an increase in the permeation flux [23]. Furthermore, the increase in the pure water flux for polymeric membranes has been attributed to the presence of hydrophilic sites in the inner walls of the filler particles as well as lower cross-linking density [23]. The results from FTIR spectra confirmed the presence of hydroxyl groups. Therefore, it can be speculated that the composite membranes have a lower packing density compared to the pure polysulfone membranes. The incorporation of the CNTs in the polysulfone matrix influences the pure water flux to varying degrees for the MMMs synthesized by adopting the three CNT dispersion methods (as confirmed by the results for the PWF). Membrane permeability is one of the requirements for a good membrane; in this study, the pure water permeability/unit length (PWP) was defined as the ratio of the PWF and the TMP. The PWP results are shown in Table 2.

The PWP at 6.9 bar was 41.7 L·m^−2^·h^−1^·bar^−1^, 159.3 L·m^−2^·h^−1^·bar^−1^, 3.7 L·m^−2^·h^−1^·bar^−1^, and 9.2 L·m^−2^·h^−1^·bar^−1^ for M1, M2, M3, and M4, respectively (see Table 2). M2 displayed the highest PWP when compared to those of M1 and M3 for the TMP range (1.38–6.9 bar) investigated in this study. M3 displayed the lowest PWP relative to the MMMs and lowest PWF, indicating a direct relationship between the PWF and the PWP. The incorporation of CNTs in the polysulfone matrix influences the permeability of the membranes as well as the pure water flux, suggesting that the CNTs play an active role on the performance of the MMMs. However, a 5% CNT loading was maintained for all the MMMs prepared using the three CNT dispersion methods during synthesis, confirming that the CNT dispersion methods also impact the performance of the MMMs. The inconsistency in the results obtained for the PWF and PWP confirms the importance of the CNT dispersion during MMM synthesis.

Homogenous dispersion of multiwalled CNTs in the polymer matrix has been reported to influence the interfacial interactions between the filler material and the polymer, ultimately affecting the performance of MMMs [10]. The presence of voids between the CNTs and the polysulfone in the MMMs could be attributed to the interfacial defects formed during synthesis, therefore influencing the permeation flux and the permeability of the membranes. Interfacial defects as a result of interface voids or rigidified polymer layers around the filler particles has been suggested to influence the permeability of MMMs by Aroon et al. [1].

#### 3.3.2. Oily Water Separation

**Figure 8:**
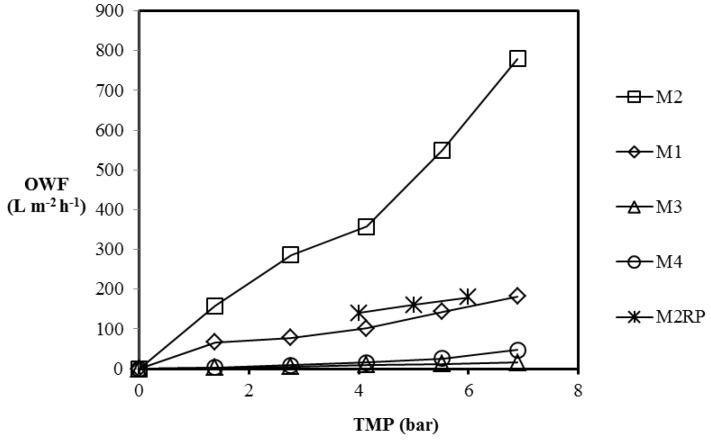
Oil–water flux (OWF) of the membranes. Experimental conditions: temperature: room temperature, membrane area: 42 cm^2^; TMP: 1.38–6.90 bar.

The emulsified oil and water mixture, with an oil concentration of 1000 mg diesel/L water, was prepared by mixing water and diesel using a high-speed stirrer for 4 h. The initial concentration of oil is typical of oilfield-produced water from oil and gas reservoirs [25]. The separation of the oil–water mixture was carried out using the Sterlitech cross-flow filtration module and the oil–water flux (OWF) was measured, with TMP varied from 1.38 bar to 6.90 bar. The effective membrane area was 42 cm^2^, the experiments were conducted at room temperature, and the fluxes were measured after attaining a steady state after 4 h. The OWF as a function of the TMP is depicted in Figure 8.

The OWF values at 6.9 bar were 182.4 L·m^−2^·h^−1^, 779.2 L·m^−2^·h^−1^, 15.6 L·m^−2^·h^−1^, and 47.6 L·m^−2^·h^−1^ for M1, M2, M3, and M4, respectively. M2 displayed the highest OWF for the given TMP range; the PWF for M2 was the highest (see Table 3). However, the OWF is lower than the PWF obtained for M2 for the given TMP range. At 6.90 bar, the OWF of the CNT/Psf membranes were in descending order: M2 > M1 > M3.

The polysulfone membrane, M4, displayed the lowest OWF when compared to those of M1 and M2, but slightly higher than that of M3. Maphutha et al. [9] reported that the OWF for their membranes are higher than that of the pure polysulfone membranes. This is in agreement with the results obtained for M1 and M2 in this study. However, the OWF obtained for M3 is not consistent with the finding from the authors, confirming further that the CNT dispersion method adopted during synthesis affects the performance of the MMMs. The PWF of M4 is higher when compared to its OWF for the TMP range, 1.38–6.90 bar. This could be attributed to hydrophobic nature of the membrane. The contact angle was measured to be 88.1° ± 2.1°, and the presence of methyl groups, which could be responsible for the hydrophobic properties of the membranes, was confirmed using the FTIR spectra as well.

The OWP at 6.9 bar was 26.4 L·m^−2^·h^−1^·bar^−1^, 113.0 L·m^−2^·h^−1^·bar^−1^, 2.3 L·m^−2^·h^−1^·bar^−1^, and 6.9 L·m^−2^·h^−1^·bar^−1^ for M1, M2, M3, and M4, respectively, as depicted in Table 3. The PWP at 6.9 bar was 41.7 L·m^−2^·h^−1^·bar^−1^, 159.3 L·m^−2^·h^−1^·bar^−1^, 3.7 L·m^−2^·h^−1^·bar^−1^, and 9.2 L·m^−2^·h^−1^·bar^−1^ for M1, M2, M3, and M4, respectively (see Table 2), confirming a decrease in the permeability of the MMMs during oil–water mixture separation when compared to the PWP. M3 displayed the lowest OWP of all the MMMs prepared in this study, and the OWP of M4 is greater than that of M3 (see Table 3). Disparities are observed in the performance of the MMMs prepared using the three CNT dispersion methods, confirmed by the MMM performance.

OWP of the membrane from Maphutha et al. [9] calculated using the reported OWF and TMP displayed a lower OWP when compared to M2 in this study. In addition, porous filler blockage has been reported to significantly reduce the performance of MMMs [1]. The lower OWP of the membranes from Maphutha et al. [9] could be attributed to the PVA layer on the CNT/Psf membrane layer.

**Figure 9:**
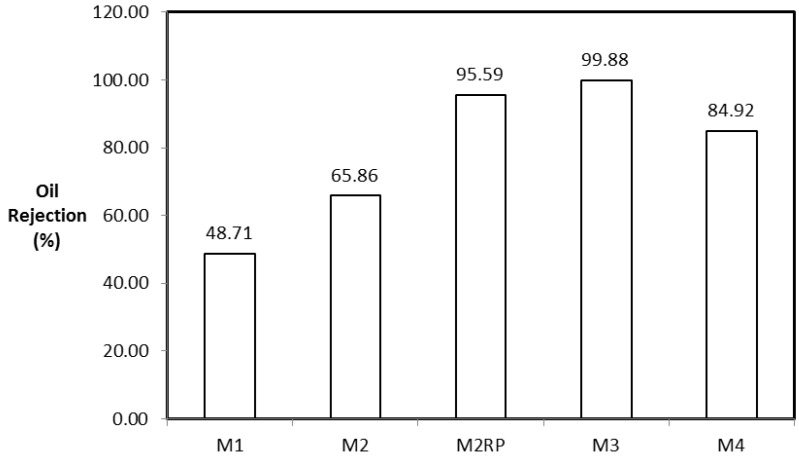
The oil rejection for the membranes. M2RP is the membrane prepared and reported by Maphutha et al. [9].

The performance of the synthesized membranes was evaluated further using the oil rejection ratio (OR) calculated using Equation (4). Samples of oil–water permeate were taken during separation tests and analyzed using a UV spectrophotometer. The oil rejection results for the membranes are depicted in Figure 9.

M3 displayed the highest OR of 99.88% when compared to the performance of other membranes (see Figure 9). The ORs for M1, M2, and M4 membranes were 48.71%, 65.86%, and 84.92%, respectively. The outstanding selectivity of the M3 membrane over those of M1, M2, and M4 membranes could be attributed to the better dispersion of CNTs in M3. The surface chemistry of the CNTs has been shown to be hydrophilic with the presence of hydroxyl groups, suggesting that CNT/Psf with uniformly dispersed hydrophilic CNTs in the polymer matrix will display a higher affinity for water as opposed to oil. Without the PVA layer on the membranes prepared by Maphutha et al. [9], it is expected that the performance of their membrane prepared using Method 2 will be similar to that of M2 in this study. The use of PVA as a layer constitutes additional membrane thickness as well as additional operating costs to the membrane fabrication. However, adopting the dispersion method, M3, in this study could alleviate the aforementioned problems without jeopardizing the expected selectivity.

The kinetic diameter of C_8_ is 7.5 Å (0.75 nm) [24] and the kinetic diameter of water is 2.6 Å (0.26 nm) [26]. Furthermore, kinetic diameter of hydrocarbons increases with increasing carbon number [27], suggesting that the components in diesel will have bigger kinetic diameters than that of C_8_. The competition between water and oil to occupy the porous sites of the membranes is clearly shown by the low OR displayed by M4, indicating that the surface chemistry of the polysulfone membranes favors the absorption of oil over water. The low OR displayed by M1 and M2 could be attributed to poor CNT dispersion and formation of interfacial defects between the CNT and polymer matrix. The increase in free fraction volume promotes permeation of oil through the membranes and hence a dramatic reduction in membrane selectivity.

Results of this study compared to literature are presented in Table 4. Despite the difference in the preparation methods and operating conditions employed in the previous studies shown in Table 4, the membrane prepared in this study and tested for the separation of oil–water emulsion displayed outstanding performance compared to that of the membranes reported.

## 4. Conclusions

Multiwalled carbon nanotubes/polysulfone composite membranes with 5% CNT loading (CNT/Psf) and pure polysulfone (Psf) membranes were synthesized using three CNT different dispersion methods. Morphology and the surface functionality of the membranes were checked with SEM and FTIR, respectively. The effect of these dispersion methods on the quality and separation performance of the membranes was evaluated during the separation of oil–water mixture. SEM micrographs depict porous cross-sections and surface morphologies for the composite membranes and polysulfone membranes; however, the degree of CNTs dispersion within the polysulfone matrix was less pronounced for composite membranes prepared using dispersion Method 2. The functional groups observed for the membranes and CNTs using FTIR spectra included carboxylic acid groups (O–H) and carbonyl group (C=O), oxygen-containing groups that have been reported to be responsible for hydrophilic properties. The contact angles measured for the MMMs using dispersion Method 1, Method 2, and Method 3 were 76.6° ± 5.0°, 77.9° ± 1.3°, and 77.3° ± 4.5°, respectively, while 88.1° ± 2.1° was measured for the pure Psf membrane (with 0% CNTs). The oil rejection displayed by the membranes prepared using dispersion Method 1, Method 2, and Method 3 was 48.71%, 65.86%, and 99.88%, respectively. The pure polysulfone membranes’ oil rejection was 84.92%. The pure water and oil water flux increased with increasing transmembrane pressure for all the membranes. The OWP of the M1, M2, and M3 membranes was 26.4 L·m^−2^·h^−1^·bar^−1^, 113 L·m^−2^·h^−1^·bar^−1^, and 2.3 L·m^−2^·h^−1^·bar^−1^, respectively. The OWP of the pure polysulfone membrane was 6.9 L·m^−2^·h^−1^·bar^−1^ at 6.9 bar. The competition between water and oil to occupy the porous sites of the membranes was confirmed by the low oil rejection for M4 when compared with M3 membranes, indicating that the surface chemistry of the membranes favors the absorption of oil over water. The low rejection observed for M1 and M2 could be attributed to poor CNT dispersion and formation of interfacial defects between the CNT and polymer matrix, resulting in free fraction volume that enhanced the permeation flux as well as the permeability. Therefore, membranes fabricated using dispersion Method 3 displayed the best performance, indicating Method 3 as the best dispersion method. The best separation performance displayed by M3 could be attributed to the efficacy of the dispersion method employed in the preparation of the precursor solution for the M3, confirming the speculation of Aroon et al. [1]. However, the synthesis protocol for M3 membranes should be optimized, as well as the operating conditions, to make the membrane fabricated via this dispersion method commercially attractive for industrial applications.

## Figures and Tables

**Table 1:** Average contact angles measured using the sessile drop method.

Membrane	Contact Angle (°)
M1	76.6 ± 5.0
M2	77.9 ± 1.3
M3	77.3 ± 4.5
M4	88.1 ± 2.1

**Table 2:** The pure water permeability/unit length (PWP) results for M1, M2, M3, and M4 using deionized water at varying TMP.

Membrane	Contact Angle (°)	PWP (L·m^−2^·h^−1^·bar^−1^)				
		1.38 bar	2.76 bar	4.14 bar	5.52 bar	6.90 bar
M1	76.6 ± 5.0	53.9	47.6	44.7	40.2	41.7
M2	77.9 ± 1.3	174.5	183.2	145.0	137.3	159.3
M3	77.3 ± 4.5	3.2	3.0	3.0	2.9	3.7
M4	88.1 ± 2.1	11.1	8.5	8.3	8.1	9.2

**Table 3:** OWP for M1, M2, M3, and M4 using deionized water.

Operating Pressure (bar)	OWP (L·m^−2^·h^−1^·bar^−1^)				
	1.38	2.76	4.14	5.52	6.90
M1	47.8	28.2	24.7	26.0	26.4
M2	114.4	103.6	86.3	137.3	113.0
M3	2.5	2.0	2.3	2.2	2.3
M4	1.9	3.0	3.7	4.7	6.9

**Table 4:** Results compared with literature.

Membrane Type	Material Treated	Filler	Oil Rejection (%)	Reference
Psf/bentonite membrane	Oil-Water mixture	Bentonite	>90	[7]
PVDF/TiO_2_/polyvinylpyrrolidone membrane	Oily wastewater	TiO_2_	99.7	[28]
Porous ceramic membrane/PVDF/PA/PVA	Oil-Water mixture	–	98.5	[8]
CNT/Psf/PVA membranes	Oil-Water mixture	CNTs	>95	[9]
CNT/Psf membranes from M3	Oil-Water mixture	CNTs	99.88	This study
